# The Increase of Simple Sequence Repeats during Diversification of Marchantiidae, An Early Land Plant Lineage, Leads to the First Known Expansion of Inverted Repeats in the Evolutionarily-Stable Structure of Liverwort Plastomes

**DOI:** 10.3390/genes11030299

**Published:** 2020-03-12

**Authors:** Jakub Sawicki, Alina Bączkiewicz, Katarzyna Buczkowska, Piotr Górski, Katarzyna Krawczyk, Patryk Mizia, Kamil Myszczyński, Monika Ślipiko, Monika Szczecińska

**Affiliations:** 1 Department of Botany and Nature Protection, University of Warmia and Mazury in Olsztyn; 10-719 Olsztyn, Poland; katarzyna.krawczyk@uwm.edu.pl (K.K.); patryk.mizia@gmail.com (P.M.); kamil.myszczynski@gmail.com (K.M.); monika.slipiko@uwm.edu.pl (M.Ś.); monika.szczecinska@uwm.edu.pl (M.S.); 2 Department of Genetics, Faculty of Biology, Adam Mickiewicz University in Poznań, 61-614 Poznań, Poland; alinbacz@amu.edu.pl (A.B.); androsac@amu.edu.pl (K.B.); 3 Department of Botany, Poznań University of Life Sciences, 60-625 Poznań, Poland; piotr.gorski@up.poznan.pl

**Keywords:** chloroplast genome, inverted repeats expansion, liverworts, cpSSR, nanopore sequencing, super-barcoding, phylogeny

## Abstract

The chloroplast genomes of liverworts, an early land plant lineage, exhibit stable structure and gene content, however the known resources are very limited. The newly sequenced plastomes of *Conocephalum, Riccia and Sphaerocarpos* species revealed an increase of simple sequence repeats during the diversification of complex thalloid liverwort lineage. The presence of long TA motifs forced applying the long-read nanopore sequencing method for proper and dependable plastome assembly, since the length of dinucleotide repeats overcome the length of Illumina short reads. The accumulation of SSRs (simple sequence repeats) enabled the expansion of inverted repeats by the incorporation of *rps*12 and *rps*7 genes, which were part of large single copy (LSC) regions in the previously sequenced plastomes. The expansion of inverted repeat (IR) at the genus level is reported for the first time for non-flowering plants. Moreover, comparative analyses with remaining liverwort lineages revealed that the presence of SSR in plastomes is specific for simple thalloid species. Phylogenomic analysis resulted in trees confirming monophyly of Marchantiidae and partially congruent with previous studies, due to dataset-dependent results of *Dumortiera*-*Reboulia* relationships. Despite the lower evolutionary rate of Marchantiales plastomes, significant barcoding gap was detected, even for recently divergent holarctic *Conocephalum* species. The sliding window analyses revealed the presence of 18 optimal (500 bp long) barcodes that enable the molecular identification of all studied species.

## 1. Introduction

The plastid genomes are the most frequently used molecules in evolutionary studies on plants, including phylogenetics, phylogeography, and population genetics [1,2,3]. They also serve as barcoding resources for most of the taxonomic groups [4], with only a few exceptions when plastid-derived barcodes fail [5,6]. In opposition to vascular plants, plastid genomes of early land plants—bryophytes are yet poorly explored.

Bryophyte plastomes, as well as well-studied angiosperm plastomes, exhibit two copies of inverted repeats (IRa and IRb) separated by two single copy regions: the large single copy (LSC) and the small single copy (SSC). In contrast to vascular plants, where plastome structure variation is observed at the family or even at genus level [7,8], in bryophytes the gene content and order seem to be very stable across evolutionary lineages. Gene content and gene order in the plastome is conserved in all main liverwort lineages, including complex thalloid, simple thalloid, and leafy species [9,10,11], despite it being millions of years since their divergence [12]. The mosses, phylogenetically sister to liverworts, are known from at least two different conformations [13] of chloroplast genome due to LSC inversion in Funariaceae [14], however the gene content of IRs, LSC, and SSC regions remained unchanged. In vascular plants of structural rearrangements that were observed within plastome is a shift in the IR boundary. The IRs of land plants fluctuate in size, either increasing the content of genes and non-coding DNA segments that otherwise would be single-copy, or losing some duplications via the transfer to single copy regions [15].

The species of genus *Conocephalum* belongs to an early divergent liverwort group—Marchantiidae characterized by thick thallus differentiated into dorsal zone (with air-chambers and air-pores) and ventral zone with storage tissue. *Conocephalum* species is one of the most common complex thallose liverwort in Holarctic, occupying wet, shaded sites in lowlands, highlands, and mountains. *Conocephalum* became the object of interest of plant science researchers, including taxonomists, geneticist, and evolutionists, due to large thalli and widespread occurrence. Biochemical followed by isoenzymatic analyses revealed the presence of cryptic speciation within *C. conicum* s.l. [16,17]. The detailed morpho-anatomical analyses resulted in the description of numerous diagnostics features enabling distinguishing *C. salebrosum* from *C. conicum* s.s. [18].

The recent phylogenetic studies revealed the lower genetic diversity of Marchantiidae plastomes in comparison to evolutionarily younger Jungermannidae [9,12], but the reasons of slower evolution of the formers are not known. The lack or low level of RNA editing in comparison to other liverworts is another characteristic feature of Marchantiidae [19,20].

In this study, we sequenced, assembled, annotated, and analyzed the plastomes of two holarctic *Conocephalum* species, which revealed unique features, including the first exception from the stable structure of liverwort plastomes. The assembly of SSR rich plastomes required the application of long-read sequencing technology, which was used for the first time in study on liverworts. Additionally, we sequenced two plastomes of early (*Sphaerocarpos texanus*) and late divergent (*Riccia fluitans*) taxa of Marchantiidae to verify whether the features that were observed in *Conocephalum* fit into any evolutionarily-wide scenario. Comparative analyses revealed species-specific SNP that are useful in molecular delimitation of *Conocephalum* species.

## 2. Results and Discussion

### 2.1. Characteristics of the Newly Sequenced Chloroplast Genomes

The short reads sequencing of prepared libraries resulted in the output data in 2–7Gb range (Table S1). The long-read nanopore sequencing was performed to provide reliable assembly over long TA-repeats regions of *Conocephalum* plastomes, resulting in over 183,000 reads (within the range 1000–83,000 bp, N50 = 2200 bp), which enabled the validation of ambiguous short-reads assembly.

**Figure 1:**
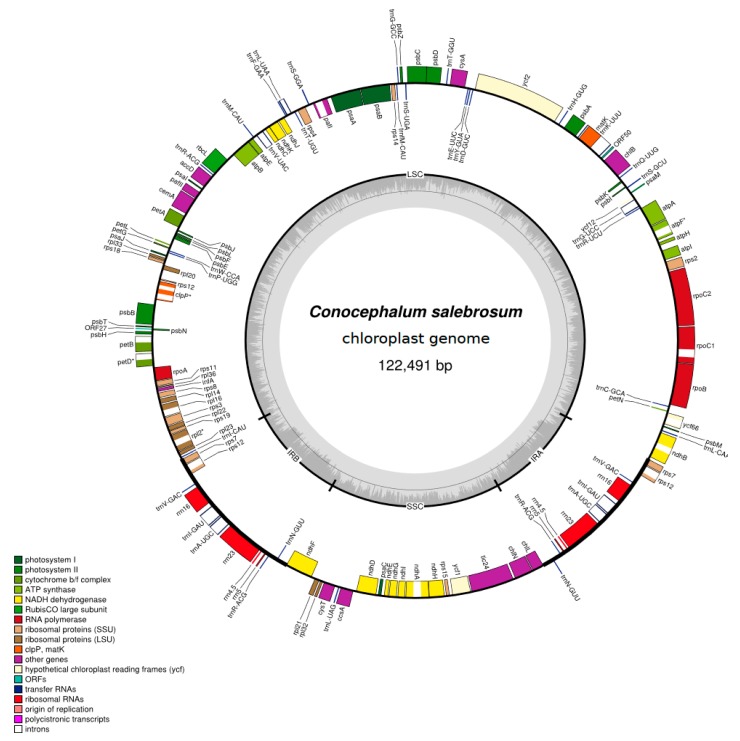
The chloroplast genome of *Conocephalum salebrosum*. Genes inside and outside the outer circle are transcribed in counterclockwise and clockwise directions, respectively. The genes are color-coded based on their function. The dashed area in the inner circle visualizes the G/C content.

The plastomes of *Conocephalum* species were 120,963 bp and 122,488 bp long in *C. conicum* and *C. salebrosum*, respectively. The structure is typical for most plants, including a pair of IR regions (each of 9612 bp in case of *C. conicum* and 11,400 bp in the case of *C. salebrosum*) separated by LSC (81,795 bp in case of *C. conicum* and 79,825 bp in case of *C. salebrosum*) and SSC (19,946  bp in the case of *C. conicum* and 19,866 bp in case of *C. salebrosum*) regions (Figure 1). 

The newly sequenced plastomes of *Riccia fluitans* and *Sphaerocarpos texanus* fall within the range with 121,999 and 121,377 bp, respectively. 

The interspecific variation in plastome length of *Conocephalum* species is higher than in the previously analysed genera with the 1525 bp difference. Comparative analysis of the chloroplast genomes of six *Aneura pinguis* cryptic species revealed that the length of the chloroplast genomes ranged from 120,698 to 121,140 bp [21], resulting in the 442 bp difference. The first sequenced liverwort plastome of *Marchantia paleacea* [22] and the later sequenced plastome of the same species differ in length by 56 bp, while the three sequenced plastomes of *M. polymorpha* differ in length by 86 bp, so the interspecific difference could be up to 860 bp, depending on the specimen. The sequenced plastomes of water-living liverwort *Riccia fluitans* differ in length by 317 bp, which is currently the biggest gap at the species level among known Marchantiales chloroplast genomes. In contrast to *Marchantia* and *Riccia* the *Conocephalum* plastomes do not reveal any intraspecific variation in length.

The enlargement of *C. salebrosum* plastome is mostly connected with the expansion of IR regions by the inclusion of two genes: *rps*12 and *rps*7. The expansion of IRs was validated with nanopore long-read sequencing and by increasing Illumina short-read coverage over duplicated *rps*12 and *rps*7 regions (Supplementary Figure S1). 

**Figure 2:**
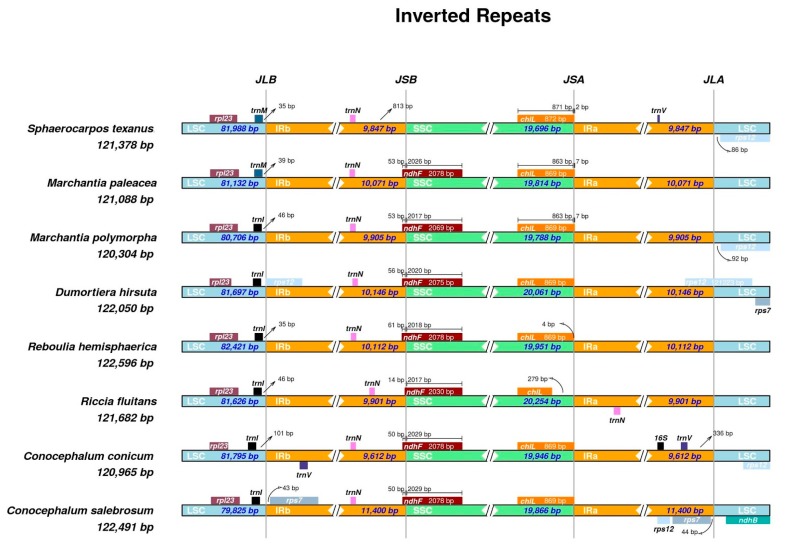
The differences in inverted repeat (IR) borders among Marchantiidae.

This is the first exception from the conservative structure of liverwort plastomes, since all of the previously sequenced ones shared the same IR, LSC, and SSR gene content. In the typical structure of liverwort plastome, *rps*12 and *rps*7 genes are located at 5′ end of LSC region between *trn*V—the last gene of IRa, and *ndh*B genes [22]. However, in the case of *C. salebrosum*, the additional copies of *rps*12 and *rps*7 are located between *trn*V-GAC (IRa) and *trn*L-CAU, which in all known liverwort plastomes is the last LSC gene at the 3’ end (Figure. 2).

The IR expansion seems to have evolved recently, since the plastome of the sister species *C. conicum* revealed a structure typical for liverworts with the IR junction presenting between *trn*V-*rpl*23 and *trn*V-*rps*12, respectively, for the IRa and IRb regions (Figure 2.). The analysed *Conocephalum* species diverged ca 15 My ago [12], therefore the IR expansion seems to be more recent than the species divergence.

Several mechanisms, such as gene conversion, double-stand breaks (DBS), and genomic deletion, are considered to be involved in the IR expansion process [23,24]. The IR expansion might be possible due to the presence of “TA” tandem repeats within the regions flanking duplicated genes, which are not present or are more reduced in other known liverwort plastomes. 

The plastomes of *Conocephalum* species contained 30% more repeats than chloroplast genomes of their relatives from genera *Marchantia*, *Dumortiera*, *Reboulia*, *Riccia*, and *Sphaerocarpos*. In comparison to the seed plants, bryophyte plastomes are richer in SSRs, with the exception of mononucleotide repeats [23], however the numbers of repeats do not seem to have an impact on structure stability by enabling the recombination. The positive correlation between the presence of SSRs and the frequency of recombination was found in human nuclear genome [24], but in the plant organellar genomes this correlation was only found in the plastomes of Geraniaceae [25]. The plastome of *C. conicum* possesses a typical structure, despite the presence of longer TA repeats.

The expansion of IRs were found in several taxa of green algae [26] and vascular plants [15], but this process characterizes rather higher taxonomic units—orders and families. Among closely related species, the gain or loss of genes by IRs is known among several genera of flowering plants [27,28], however, for non-flowering plants, the differences in IR gene content at the genus level are reported for the first time.

### 2.2. Phylogenetics Relationships Based on Plastomes Datasets

The reliable alignment of complete genomes is difficult or impossible to obtain due to the high divergence of intergenic and intronic regions of liverwort plastomes, therefore the main phylogenetic analyses were split into two datasets: the first one included coding regions (CDS and ribosomal genes) of all liverwort lineages, while the second one, based on complete genomes, only comprised complex thalloid species.

**Figure 3:**
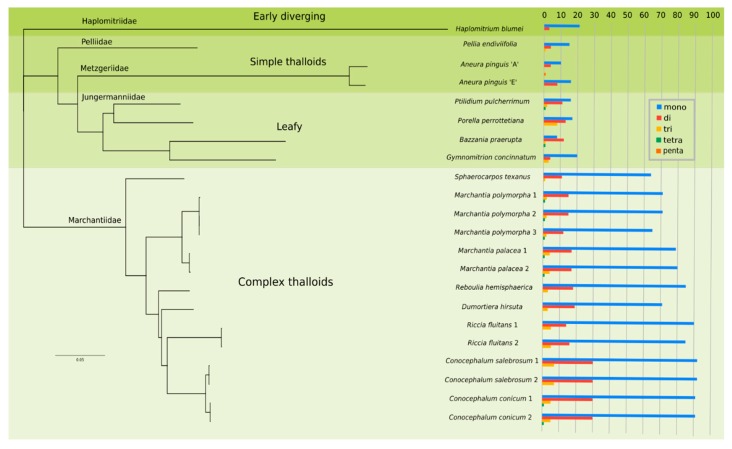
Phylogram based on coding regions of liverworts plastomes derived from a Bayesian analysis. The posterior probabilities values were equal to 1 for each of the clade. Right side of the figure provides data on SSR abundance in liverworts plastomes.

The analysed dataset that was based on CDS and ribosomal genes delivered results that were mostly congruent with previous phylogenetic studies [12,19,20,29] and divided liverworts into five main lineages: Haplomitriidae, represented here by *Haplomitrium blumei*, which served as an outgroup, early divergent complex thalloids, followed by two lineages of simple thalloid and leafy liverworts (Figure 3)

**Figure 4:**
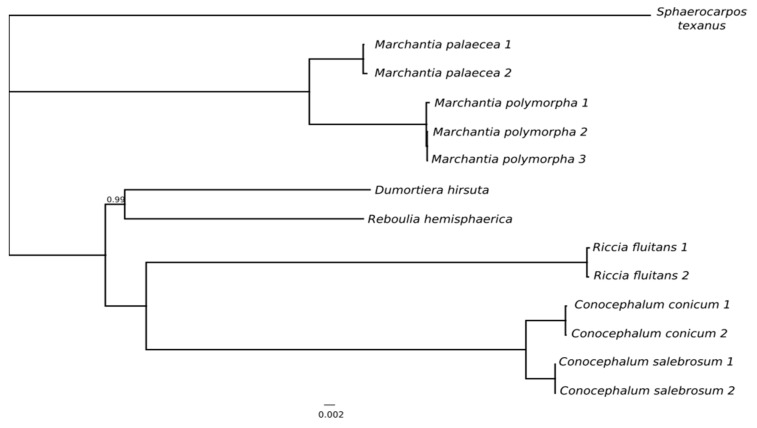
Phylogram of Marchantiidae derived from a Bayesian analysis of complete large single copy (LSC) and small single copy (SSC) regions. The posterior probabilities values lower than 1 are shown. The IR region was excluded from analysis due to incongruent phylogenetic signal.

Bayesian and Maximum Likelihood analyses both provided the trees with identical topology and clade support. All of the groups mentioned above formed distinct clades with maximum bootstrap support and Bayesian posterior probabilities. Phylogeny that is based on the complete set of plastome coding regions is congruent with previous studies [12,29], however phylogenetic analysis of Marchantiales based on complete chloroplast genomes revealed incongruence with previously published data due to the position of *Dumortiera hirsuta* and *Reboulia hemisphaerica*. In the latest, most complex study of Marchantiales using 11 loci from three genomes (one nuclear, three mitochondrial, and seven plastid), *Dumortiera* was resolved as an earlier divergent than *Reboulia* [12], while the results that were based on complete plastid genomes placed these species in a common clade (Figure S2), however weakly (58% of ML bootstrap support) or unsupported (in case of Bayesian analysis). The analysis of complete genomes, including spacers and introns, did not provide satisfactory resolution of *Reboulia*-*Dumortiera* relationships (Figure S2). The decrease of phylogenetic resolution due to the expansion of the dataset by spacers and introns is rather uncommon, since non-coding regions are usually more variable. However, the barcoding analyses revealed that phylogenetic incongruence is accumulated in the IR region of plastome, therefore additional analysis of single copy plastome regions were performed. The exclusion of IR region did not change the topology of trees (Figure 4), but provided significant (0.99 of Bayesian inference) and reasonable (70% ML BS) support for the *Reboulia*-*Dumortiera* clade. The monophyly of this clade was also reported in recent studies providing results that were based on mitogenomic data [19].

### 2.3. Mining and Characterisation of SSRs in Liverwort Plastomes

The liverwort plastomes were previously considered as SSR rich, especially in comparison with tracheophytes, however this conclusion had been made based on very limited data, including only *Marchantia* [23]. Analysis of newly sequenced and available plastomes of liverworts performed in this study revealed a wide range of SSRs with different accumulation patterns (Figure 3), which only confirmed the previous observations in the case of Marchantiidae [23]. The species belonging to Marchantiidae revealed a high number of repeats, especially mono- and dinucleotides, however longer motifs, including tri-, tetra-, and pentanucleotide repeats, were too rare to draw any conclusion. The number and length of mononucleotide repeats present in the analysed plastomes of complex thalloids was rather stable. The number of repeats ranged from 64 in the early divergent *Sphaerocarpos* to 92 in *C. salebrosum* (Figure 3, Table S1) and their length (Table S2) fell into the range from 13 (*C. salebrosum*) to 17 (*Conocephalum conicum*, *Marchantia paleacea*, *Sphaerocapos texanus*).

The chloroplast genomes of *Conocephalum* species revealed a stable number of SSRs, the highest among liverworts (91–92 mono-, 30–32 dinucleotide), but they were very diverse in terms of their length. The longest dinucleotide motif that was observed in *C. conicum* was almost two times longer than in *C. salebrosum* (63 vs 33 AT repeats). Therefore, the reliable assembly of *C. conicum* plastome had to be supported with nanopore long-read sequencing.

Among the *Marchantia* species the interspecific differences were noticed in the case of mono-, di-, and trinucleotide repeats and, in all cases, the greatest number of SSRs was found in *M. paleacea* (Figure 3, Table S1). The tri- and tetranucleotide repeats were 75% longer in *M. paleacea,* albeit the length of mono- and dinucleotide SSRs was similar among the analysed species. The higher intrageneric variation of SSR present in *Marchantia*, could be explained by the more than twice longer regions in comparison to relatively younger species of *Conocephalum* [12]. The three available plastomes of *Riccia fluitans* revealed the highest variation in the numbers of mononucleotide repeats that fall within the range of 85 to 93.

The plastome of *Dumortriera hirsuta*, sister to *Marchantia* genus, contain a similar number of SSRs, while the basal for Marchantiidae, *Sphaerocarpos texanus* were characterized by the lowest number of identified repeats. The trend of SSR cumulation during complex thalloid diversification is clearly visible, despite the limited data (Figure 3). 

The plastomes of Jungermanniales revealed a smaller number of repeats (Figure 3, Table S1), similar to those that were found in vascular plants [23], so the previously published conclusion should be narrowed only to complex thalloid liverworts. The SSR-gaining scenario is also supported by the low numbers of SSRs in the early divergent liverwort *H. blumei*, where only 24 SSRs were found including 21 mononucleotide and three dinucleotide repeats (Figure 3). 

### 2.4. Plastome Diversity and Molecular Delimitations of Marchantiales

**Figure 5:**
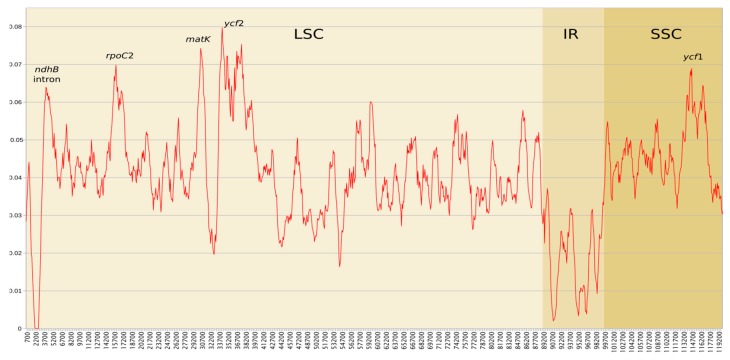
Nucleotide diversity (Pi) by sliding window analysis in the aligned whole plastomes (excluding second IR) of 14 Marchantiidae specimens. Window length 1000 bp, step size 100bp.

The dataset containing 14 complete sequences of chloroplast genomes revealed relatively low level of genetic diversity. Similar to other taxonomic groups [30,31,32], the lowest mean nucleotide diversity (0.0182) was detected in the IR region, while the single copy regions were more diverse, with Pi values of 0.0415 and 0.0453 for LSC and SSC, respectively (Figure 5). 

The IR that were located genes and spacers are known from their lower substitution rates in comparison to those located in single copy parts of plastomes [6,7,13,33]. Genes that are transferred from SC regions to IRs tend to decrease substitution rates due to copy-dependent repair activity [13,34]. Lower nucleotide diversity of IR regions is also observed in liverworts and mosses, but available data are still very limited [21,31]. The comparative analysis of six *Aneura* plastomes revealed 4.6-fold decrease of IR nucleotide diversity in comparison to single copy regions [21]. Similar values were also obtained from plastomes of Orthotrichaceae moss family [31]. The plastomes of Marchantiidae that were sequenced and analysed in this study revealed lower nucleotide diversity than among species of *Aneura* genus, however the Marchantiidae are known from low evolutionary rates of their organellar genomes [12]. In case of genes *rps*7 and *rps*12 that are transferred to IR of *C. salebrosum* plastome it is too early to conclude whether this transfer influences their substitution ratio, since the plastome sequences of only four individuals are known. In the single copy regions, the highest value was found for *ycf*2 gene (pi = 0.008), which is often considered as a promising barcode in many other taxa [7,35]. The second (*matK*) and third (*rpoC*2) most variable regions of Marchantiidae plastome also prove their potential as barcodes in many plant groups and are among most commonly used regions in plant barcoding [36,37,38,39]. The remaining regions with pi > 0.006, the *ndh*B intron in LSC and *ycf*1 gene in SSC were also previously considered as potential barcodes in plants [40,41].

In the case of five species from three genera (*Conocephalum*, *Marchantia* and *Riccia*), at least two individuals were sequenced, thus enabling barcoding analyses, which revealed the presence of significant barcoding gap for all the species (Figure. S3). 

**Figure 6:**
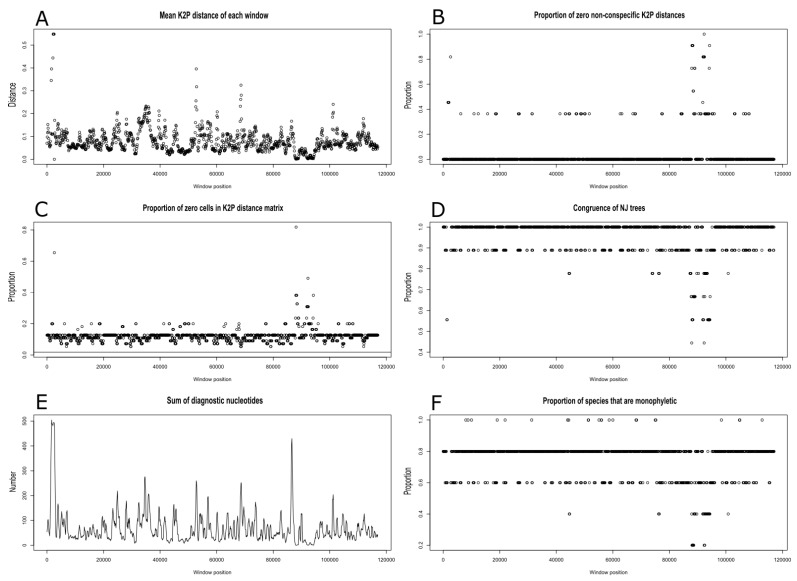
Results of several analyses across the plastome sequences of five Marchantiidae species with at least two sequenced specimens using the sliding window method. (**A**)—the plot of the mean Kimura 2-parameter distance matrix for each 500 bp-long window. (**B**)—the proportion of zero non-conspecific distances. (**C**)—the proportion of zero cells in the distance matrix. The unbroken horizontal line crossing the y-axis at 0 is the proportion of zero cells in the distance matrix created from the full dataset. (**D**)—the proportion of clades that are identical between the windows and the full dataset. (**E**)—the sum of diagnostic nucleotide positions for all species. (**F**)—the proportion of species that are monophyletic.

The highest interspecific distances were detected in the case of *Riccia fluitans* (K2P distance 9.7 percent) with *Marchantia* in the middle (K2P distance ranged from 2.55 to 2.58 percent) and the lowest in the case of *Conocephalum* species (K2P distance 1.05 percent). These results correspond well with the estimated divergence times of *Marchantia* and *Conocephalum* species, which are 40 Mya and 15 Mya, respectively [12]. The sliding window analyses across plastomes revealed the lowest K2P distances and the lowest numbers of diagnostic nucleotides across the IR region (Figure 6A,E). The IR region was also characterised by the greatest number of zero cell in K2P distance matrix and the highest proportion of zero non-conspecific K2P distances (Figure 6B,C). The above parameters are also correlated with the disturbance of phylogenetic signal at the IR regions, which is the main source of the incongruent NJ trees (Figure 6D). None of 18 detected 500 bp frames that enable molecular delimitation of all analysed species were found in IR, three of them were located in SSC, while also remaining in LSC regions (Figure 6F).

The genetic diversity of complex thalloid liverwort plastomes seems to be lower in comparison to simply thalloid and leafy taxa [12]; however, available genomic resources are still very limited. The complete plastome sequences are known from the four genera of complex thalloids, including *Marchantia* [42], *Dumortiera* [43], *Reboulia*, *Riccia* [29], and four species sequenced in this study. The sequenced genomes of simple thallioids are limited to genera *Pellia* [44] and *Aneura* [21,45], while the sequenced complete leafy liverwort plastomes comprise 14 species [9,10,29].

## 4. Materials and Methods 

### 4.1. DNA Extraction

The total genomic DNA of liverworts were extracted from fresh tissue stored in−20 °C. The DNA was extracted using the Qiagen Plant MiniSpin (Qiagen, Germany). Ca 1 cm^2^ of cleaned thallus was ground with silica beads in a MiniBead-Beater homogenizer for 50 s and subsequently processed according to the manufacturer protocol. The DNA of *Riccia fluitans* and *Sphaerocarpos texanus* were extracted from 1 cm long thalli from herbarium specimens.

The DNA quantity was estimated using Qubit fluorometer and Qubit™ dsDNA BR Assay Kit (Invitrogen, Carsbad, NM, USA). DNA quality was checked by electrophoresis in 0.5% agarose gel that was stained with Euryx Simple Save (Eurx, Gdańsk, Poland). Table S1 provides sample specimen details.

The extracted DNA of *Conocephalum* species prior to nanopore sequencing was carefully examined and additionally cleaned while using Genomic DNA Clean and Concentrator kit (Zymo, Irvine, CA, USA).

### 4.2. Library Construction and Sequencing

The libraries for short-read sequencing were prepared using Qiagen FX library preparation kit according to the manufacturer protocol and sequenced while using HiSeq X platform (Illumina) by Macrogen Inc. (Seoul, Korea) to generate 150 bp paired-end reads with a 350 bp insert between paired reads.

The long-read libraries were constructed using Rapid sequencing kit RAD-004 (Oxford Nanopore, UK), according to manufacturer’s protocol and sequenced using the MinION MK1b portable device (Oxford Nanopore) with default settings and Guppy basecaller for improved read accuracy.

The nanopore reads were polished using the hybrid read error correction method in order to use sequencing reads of the best quality. First, the Burrows–Wheeler Transform (BWT) of the short-read Illumina dataset was constructed while using ropeBWT2 [46]. Next, FMLRC [47] was used to build the FM-index and correct errors occurring within nanopore reads.

### 4.3. Genomes Assembling, Annotation and Comparative Analyses

Sequencing reads were cleaned by removing the adaptor sequences and low-quality reads with Trimmomatic v0.36 [48] and assembled while using SPAdes 3.12.0 [49]. Reference plastome sequences of *Marchantia polymorpha* (NC035977.1) were used to identify the organellar genomes of Marchantiidae among the generated contigs. The detailed workflow was similar to that previously published [50]. 

The initial assemblies of *Conocephalum* plastomes revealed several long TA-rich regions, which made reliable assembly using only short reads difficult, therefore the nanopore sequencing was applied to confirm the assumed linkage between contigs flanking by “TA” motifs. The polished nanopore reads were mapped on Illumina reads based contig flanked by microsatellites motifs using Geneious Prime 2019 build-in mapper with high-sensitivity settings and minimum overlap set to 2000 bp. The consensus sequenced with nanopore overlaps longer than flanking SSR enabled the proper orientation of input contigs. The obtained circular assemblies were revalidated by mapping short-reads libraries in Geneious Prime 2019.

Annotated organellar genomes GenBank files were used to draw gene maps while using the OrganellarGenome DRAW 1.31 tool [51], and the maps were examined for further comparisons of gene order and content.

The four junctions between single-copy segments and inverted repeats in *Riccia fluitans* and *Sphaerocapros texanus* were confirmed using PCR-based product sequencing of the assembled genomes. Supplementary File 1 provides the amplifications conditions, used reagents, and primers. Purified PCR products were sequenced in both directions using the ABI BigDye 3.1 Terminator Cycle Kit (Applied Biosystems, Foster City, CA, USA) and visualized with the ABI Prism 3130 Automated DNA Sequencer (Applied Biosystems). The sequences that were obtained with the Sanger method were aligned with the assembled genomes using the Geneious Prime 2019 assembly software to check for any differences.

The chloroplast microsatellite markers were identified using the msatcommander application [52] with default settings.

### 4.4. Phylogenomics Analyses

Phylogenetic analyses were performed using the chloroplast genomes of 32 species, including all known and newly sequenced plastomes, complex thalloids, and selected species of remaining liverworts lineages. MAFFT software [53] was used to create an alignment of LSC, IR, and SSR chloroplast regions. Next, with the use of PartitionFinder2 [54], the best partitioning schemes and corresponding substitution models of the alignment were estimated. Afterwards, Bayesian and ML analyses were conducted using MrBayes 3.2.6 [55] and RaxML [56] software, respectively, based on the alignment and obtained models. The MCMC algorithm was run for 5,000,000 generations (sampling every 500) with four incrementally heated chains (starting from random trees). The Tracer 1.7.1 [57] software was used to determine the number of generations needed to reach stationarity, which occurred at approximately 300,000 generations. Therefore, the first 600 trees were discarded as burn-in, and the remaining trees were used to develop a Bayesian consensus tree. The ML analysis was performed with the partitioning scheme and nucleotide substitution blocks that were generated by PartitionFinder2, using default parameters. Bootstrap analyses were performed with 2000 replicates to assess the nodal support. The phylogenetics analyses were conducted while using three datasets: the coding regions of plastomes for all of the analysed liverworts, complete plastome sequences of Marchantiidae (since its unable to provide reliable alignment of intergenic regions among phylogenetically distant liverworts lineages), and the dataset containing complete single copy regions of Marchantiidae. The latter was performed due to the detected incongruence in phylogenetic signal among single copy regions and inverted repeats. 

### 4.5. Diversity Analyses

Available and newly sequenced Marchantiidae chloroplast genomes were aligned using the MAFFT v5 genome aligner [53]. Visually checked alignments were imported in the R environment and analysed using the PopGenome package [58]. The nucleotide diversity (Pi) was calculated for each of the plastome region (LSC, IR and SSC) and using a sliding window approach with 1000 bp window length and 100 bp step. Barcoding analyzes of *Conocephalum*, *Marchantia* and *Riccia* plastomes were performed in Spider software [59] using 500 bp-long sliding window with 100 bp step.

## 5. Conclusions

The result of our study revealed the increase of SSR during the diversification of complex thalloid liverworts, which, in the case of SSRs located near IR-LSC boundaries, leads to the expansion of IRs via homologous recombination. The growing length and number of dinucleotide repeats, which often override the length of commonly used Illumina 100–150 bp long reads, is pushing towards the new long-read sequencing technologies, like nanopore sequencing, which enable reliable structural assembly of repeat-rich molecules.

The newly sequenced genomes confirmed the stable structure of liverwort plastomes, with one exception for *Conocephalum salebrosum*, where the significant expansion of IRs was detected for the first time. This expansion might be the result of SSR accumulation during the diversification of Marchantiidae. Despite being SSR rich, the plastomes of complex thalloids are less diverse than other liverwort lineages. The low genetic diversity of Marchantiales chloroplast genomes does not exclude their use as a good source of barcodes. Screening for potential barcodes revealed 18 optimal, 500 bp long frames. Each of them enables the molecular identification of all the analysed taxa. The presented study also points out another important aspect of phylogenetics studies—the larger datasets based on complete genomes do not always resolve evolutionary relationships better. In this particular case, an incongruent phylogenetic signal came out from slowly evolving inverted repeat regions.

## Supplementary Materials

The following are available at https://www.mdpi.com/2073-4425/11/3/299/s1. Table S1: The list of specimens used in this study including sequencing results and numbers of SSRs. Table S2: The number and length of SSRs detected in analyzed plastomes, Supplementary File 1: The conditions of PCR reaction used during study, Figure S1: Read coverage LSC-IR border of *Conocephalum salebrosum* chloroplast genome. Figure S2: Phylogram of Marchantiidae derived from a Maximum Likelihood analysis of complete chloroplast sequences. The posterior probabilities values lower than 1 and bootstrap values lower than 100% are shown. The Bayesian analysis resulted in the tree with identical topology but unsupported (0.72 posterior probability) *Dumortiera-Reboulia* clade. Figure S3: Line plot of the barcode gap for the five species of Marchantiales. For each individual in the dataset, grey lines represent the furthest intraspecific distance (bottom of line value), and the closest interspecific distance (top of line value).

## Abbreviations

LSC	Large Singe Copy Region
SSC	Small Single Copy Region
IR	Inverted Repeats Regions
Bp	Base Pair
BS	Bootstrap Support
SSR	Simple Sequence Repeats
ML	Maximum Likelihood
MCMC	Monte Carlo Markov Chains
NJ	Neighbour-Joining
