# Supplementary figures and images for: The Increase of Simple Sequence Repeats during Diversification of Marchantiidae, An Early Land Plant Lineage, Leads to the First Known Expansion of Inverted Repeats in the Evolutionarily-Stable Structure of Liverwort Plastomes
Source: Genes (Basel). 2020 Mar 12;11(3):299. doi: 10.3390/genes11030299 (PMC7140840; doi:10.3390/genes11030299)

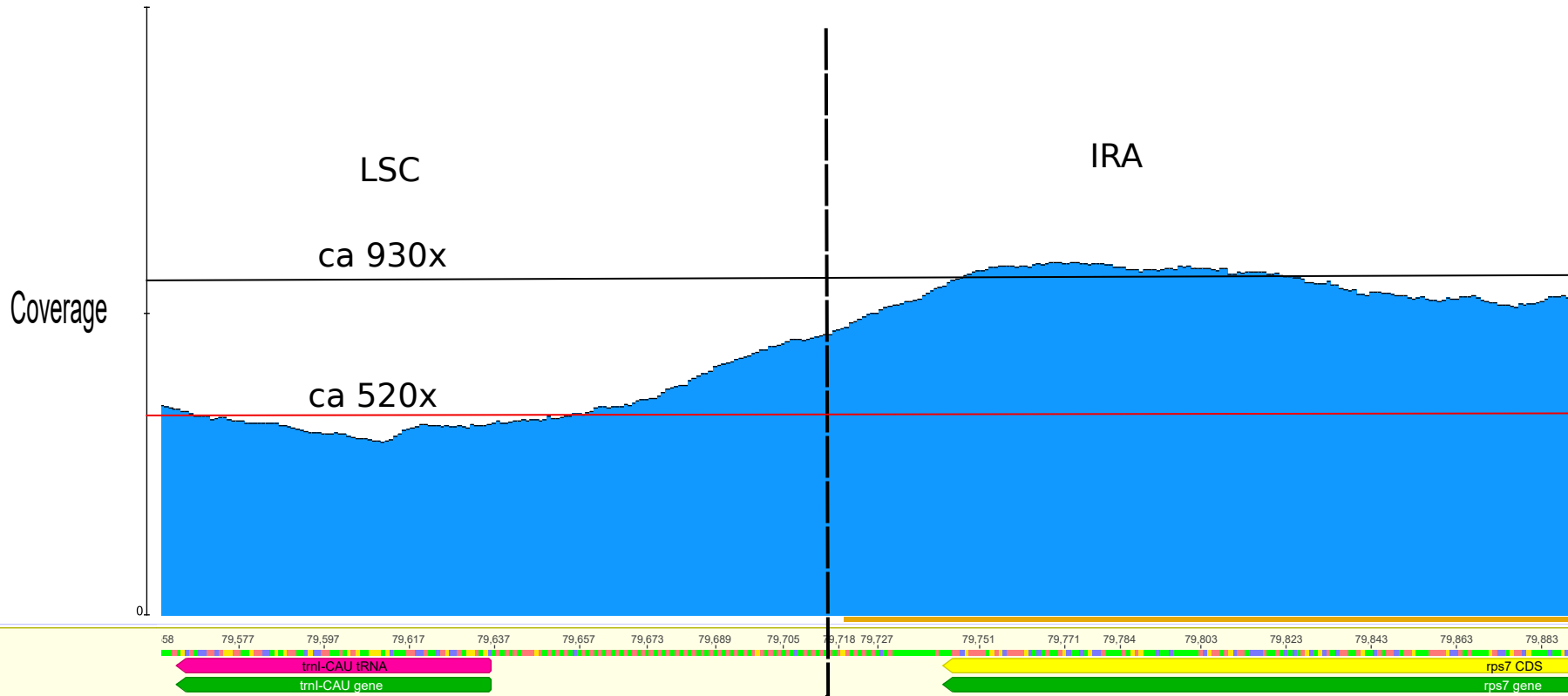

Supplement: Supplementary file 1 [file genes-11-00299-s001.zip › supplementary figure 1.pdf]

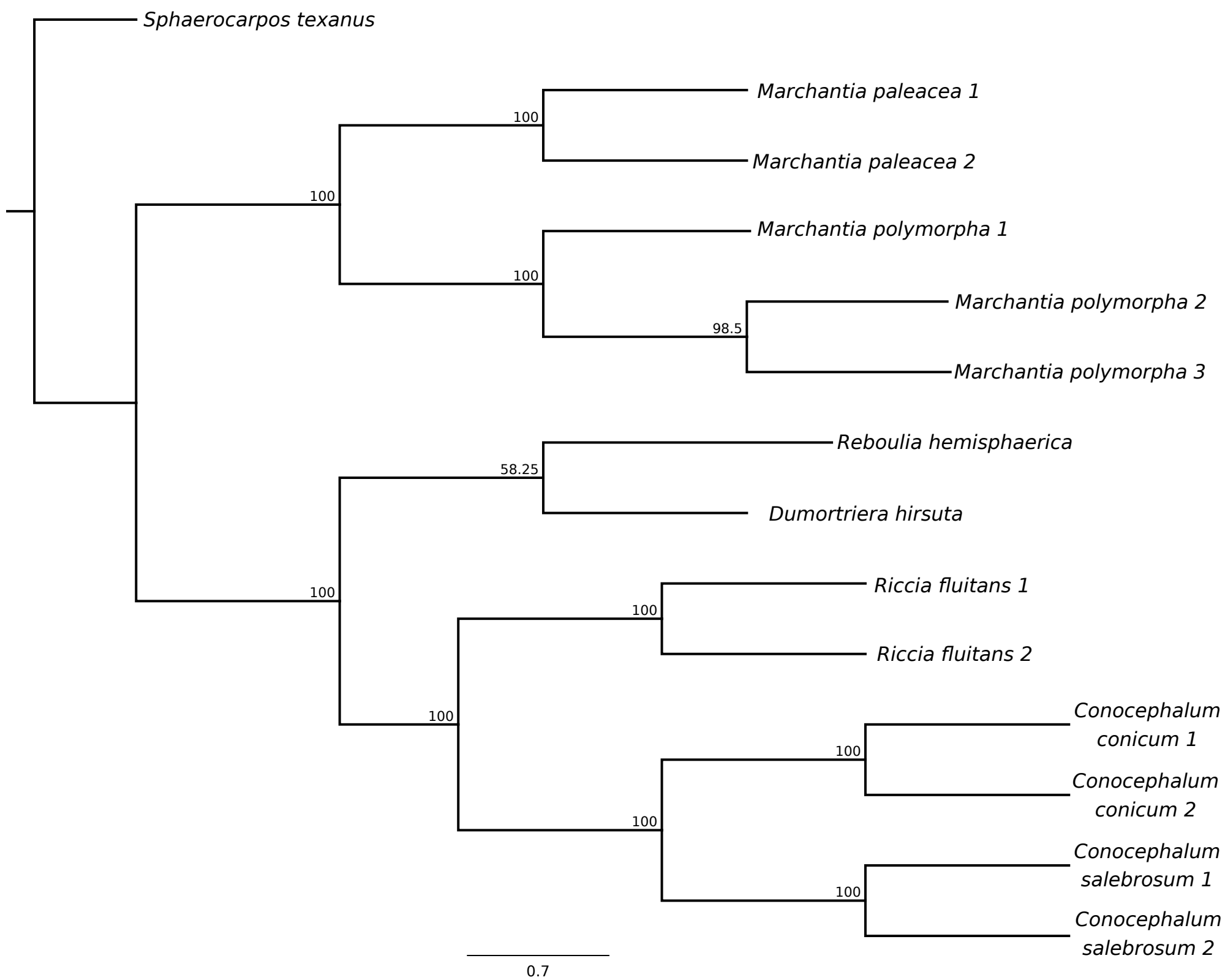

Supplement: Supplementary file 1 [file genes-11-00299-s001.zip › supplementary figure 2.pdf]

Percent K2P distance

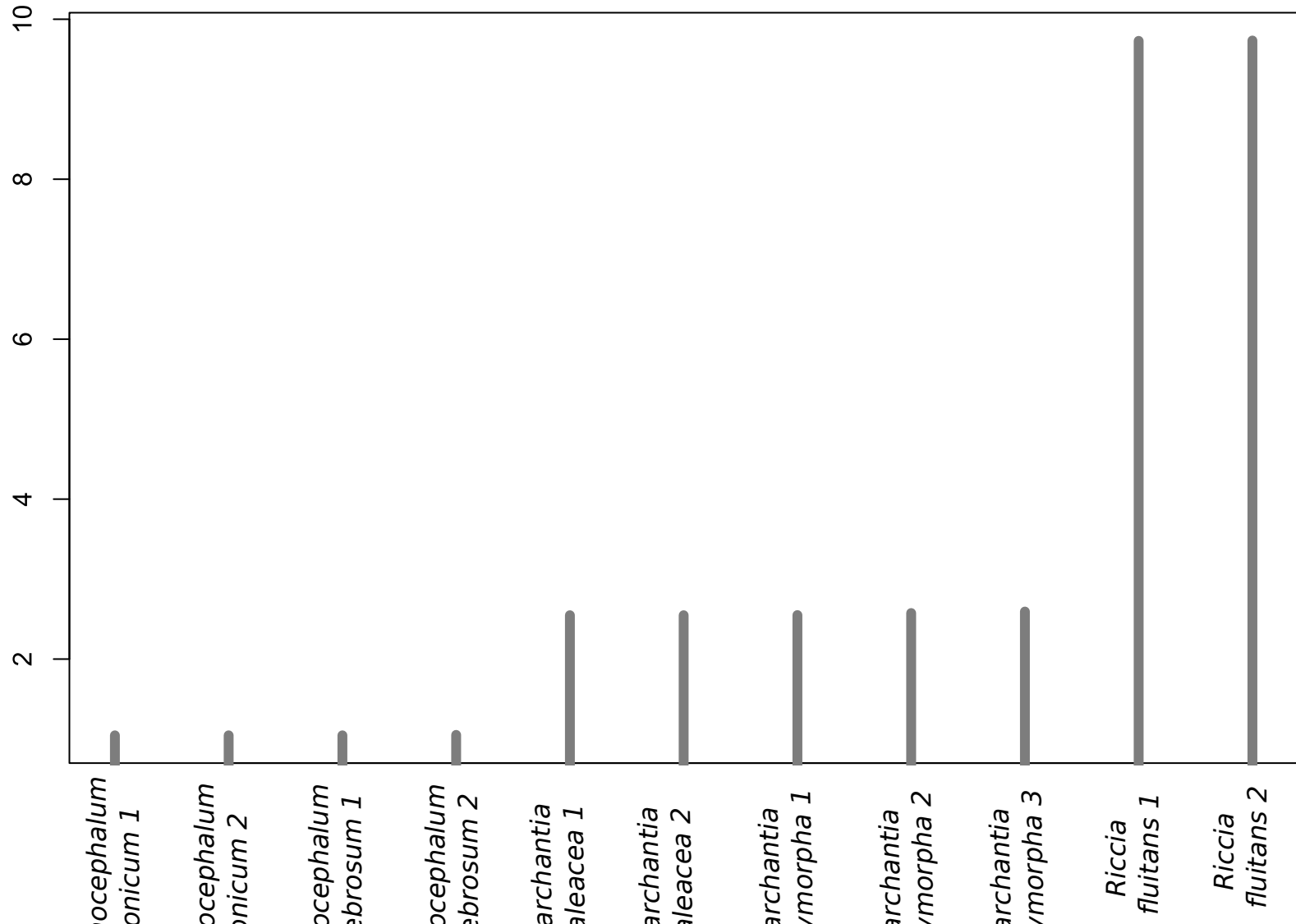

Supplement: Supplementary file 1 [file genes-11-00299-s001.zip › supplementary figure 3.pdf]
