# Supplementary material for: The Increase of Simple Sequence Repeats during Diversification of Marchantiidae, An Early Land Plant Lineage, Leads to the First Known Expansion of Inverted Repeats in the Evolutionarily-Stable Structure of Liverwort Plastomes
Source: Genes (Basel). 2020 Mar 12;11(3):299. doi: 10.3390/genes11030299 (PMC7140840; doi:10.3390/genes11030299)
Supplement: Supplementary file 1 [file genes-11-00299-s001.zip › Supplementary file 1.doc]

The junction regions between IR, LSC and SSC were confirmed using PCR. Four primers pairs were designed based on the conserved Marchantiidae nucleotide sequences that overlap edges of four LCBs pairs. The sequences of primers were as follow:

**LSC – IRa**: Forward – CTTTAACCATTCAGCCATGGATGCT

Reverse - ACTCTACCGCTGAGTTATATCCCT

**IRa-SSC**: Forward - TTAGCCCATAAACTGTTCAAAGAAC

Reverse - AGCCAAAGACTCTACAACATGAAG

**SSC-IRa** – Forward – TTCCATAAACTGCTATTTTCATA

Reverse - CCACTGAGCTACTGAGGAACAATG

**IRa-LSC**: Forward - CTACCGCTGAGTTATATCCCTAC

Reverse - TTGGTGTTGTAGTGGAAAAGTTGA

PCR reactions were performed in 25 μL of a reaction mixture containing 20 ng of DNA, 1x PCR buffer, 1.5 mM MgCl_2_, 200 μM dNTP (dATP, dGTP, dCTP, dTTP), 1.0 μM of each primer and 1 U RUN polymerase (A&A Biotech, Gdańsk, Poland). Reactions were performed under the following thermal conditions: (1) initial denaturation—4 min at a temperature of 94 °C; (2) denaturation—45 s at 94 °C; (3) annealing—50 s at 56 °C for, (4) elongation–60 s at 72 °C; (5) final elongation—7 min at 72 °C. Stages 2–4 were repeated 35 times.
